# Supplementary material for: Corporate governance, financial performance, and economic policy uncertainty. Evidence from emerging Asian economies
Source: PLoS One. 2024 Dec 5;19(12):e0312292. doi: 10.1371/journal.pone.0312292 (PMC11620350; doi:10.1371/journal.pone.0312292)
Supplement: Attachment — Submitted filename: Plos One Review....pdf [file pone.0312292.s003.pdf]

PLOS ONE

Corporate Governance, Financial Performance and Economic Policy Uncertainty: Evidence From Emerging Asian Economies

--Manuscript Draft--

**Manuscript Number:**	PONE-D-23-36973
**Article Type:**	Research Article
**Full Title:**	Corporate Governance, Financial Performance and Economic Policy Uncertainty: Evidence From Emerging Asian Economies
**Short Title:**	Role of Governance and Policies in Economy
**Corresponding Author:**	Chaoyang Zhang<br>Henan Institute of Economics and Trade<br>Zhengzhou, CHINA
**Keywords:**	corporate governance; Financial Performance; Economic Policy Uncertainty; And Emerging Economies
**Abstract:**	Objective of this study is to examine the impact of corporate governance and economic policy uncertainty on financial performance in non-financial firms in three Asian emerging economies, Pakistan, China, and Malaysia. The study analyzed 400 non-financial firms listed on the stock markets of these countries for a period of 10 years (2012-2021). Data on corporate governance, financial performance, and CSR scores were obtained from DataStream. The study used regression analysis and the generalized method of moments (GMM) for its robustness analysis. Our findings show that all attributes of the corporate governance practices have significant positive impact on return on asset except existence of audit board committee with respect to all selected economies. Moreover, corporate governance practices have a significant positive relationship with return on equity. However, in the case of EPS all attributes are significant positive relationship except board size with earning per share. The economic policy uncertainty has significant moderating impact between corporate governance practices and financial performance of organizations belongs to Asian economies. This study advocated the implications for the government and policy makers to improve the corporate governance practices especially during the period of high economic uncertainty.
**Order of Authors:**	Chaoyang Zhang
**Additional Information:**	
**Question**	**Response**
**Financial Disclosure**<br><br>Enter a financial disclosure statement that describes the sources of funding for the work included in this submission. Review the submission guidelines for detailed requirements. View published research articles from *PLOS ONE* for specific examples.<br><br>This statement is required for submission and **will appear in the published article** if the submission is accepted. Please make sure it is accurate.	The author(s) received no specific funding for this work.

Powered by Editorial Manager® and ProduXion Manager® from Aries Systems Corporation

**Funded studies**<br>Enter a statement with the following details:<br>• Initials of the authors who received each award<br>• Grant numbers awarded to each author<br>• The full name of each funder<br>• URL of each funder website<br>• Did the sponsors or funders play any role in the study design, data collection and analysis, decision to publish, or preparation of the manuscript?<br><br>Did you receive funding for this work?	
**Competing Interests**<br><br>Use the instructions below to enter a competing interest statement for this submission. On behalf of all authors, disclose any competing interests that could be perceived to bias this work—acknowledging all financial support and any other relevant financial or non-financial competing interests.<br><br>This statement is required for submission and **will appear in the published article** if the submission is accepted. Please make sure it is accurate and that any funding sources listed in your Funding Information later in the submission form are also declared in your Financial Disclosure statement.<br><br>View published research articles from *PLOS ONE* for specific examples.	The authors have declared that no competing interests exist.

Powered by Editorial Manager® and ProduXion Manager® from Aries Systems Corporation

**NO authors have competing interests**<br><br>Enter: *The authors have declared that no competing interests exist.*<br><br>**Authors with competing interests**<br><br>Enter competing interest details beginning with this statement:<br><br>*I have read the journal's policy and the authors of this manuscript have the following competing interests: [insert competing interests here]*<br><br>* typeset	
**Ethics Statement**<br><br>Enter an ethics statement for this submission. This statement is required if the study involved:<br><br>• Human participants<br>• Human specimens or tissue<br>• Vertebrate animals or cephalopods<br>• Vertebrate embryos or tissues<br>• Field research<br><br>Write "N/A" if the submission does not require an ethics statement.<br><br>General guidance is provided below. Consult the submission guidelines for detailed instructions. **Make sure that all information entered here is included in the Methods section of the manuscript.**	N/A

**Format for specific study types**<br><br>**Human Subject Research (involving human participants and/or tissue)**<br>• Give the name of the institutional review board or ethics committee that approved the study<br>• Include the approval number and/or a statement indicating approval of this research<br>• Indicate the form of consent obtained (written/oral) or the reason that consent was not obtained (e.g. the data were analyzed anonymously)<br><br>**Animal Research (involving vertebrate animals, embryos or tissues)**<br>• Provide the name of the Institutional Animal Care and Use Committee (IACUC) or other relevant ethics board that reviewed the study protocol, and indicate whether they approved this research or granted a formal waiver of ethical approval<br>• Include an approval number if one was obtained<br>• If the study involved *non-human primates*, add *additional details* about animal welfare and steps taken to ameliorate suffering<br>• If anesthesia, euthanasia, or any kind of animal sacrifice is part of the study, include briefly which substances and/or methods were applied<br><br>**Field Research**<br><br>Include the following details if this study involves the collection of plant, animal, or other materials from a natural setting:<br>• Field permit number<br>• Name of the institution or relevant body that granted permission	
**Data Availability**<br><br>Authors are required to make all data underlying the findings described fully available, without restriction, and from the time of publication. PLOS allows rare exceptions to address legal and ethical concerns. See the PLOS Data Policy and FAQ for detailed information.	Yes - all data are fully available without restriction

A Data Availability Statement describing where the data can be found is required at submission. Your answers to this question constitute the Data Availability Statement and **will be published in the article**, if accepted.<br><br>**Important:** Stating 'data available on request from the author' is not sufficient. If your data are only available upon request, select 'No' for the first question and explain your exceptional situation in the text box.<br><br>Do the authors confirm that all data underlying the findings described in their manuscript are fully available without restriction?	
**Describe where the data may be found in full sentences. If you are copying our sample text, replace any instances of XXX with the appropriate details.**<br><br>• If the data are **held or will be held in a public repository**, include URLs, accession numbers or DOIs. If this information will only be available after acceptance, indicate this by ticking the box below. For example: *All XXX files are available from the XXX database (accession number(s) XXX, XXX.*).<br>• If the data are all contained **within the manuscript and/or Supporting Information files**, enter the following: *All relevant data are within the manuscript and its Supporting Information files.*<br>• If neither of these applies but you are able to provide **details of access elsewhere**, with or without limitations, please do so. For example:<br><br>*Data cannot be shared publicly because of [XXX]. Data are available from the XXX Institutional Data Access / Ethics Committee (contact via XXX) for researchers who meet the criteria for access to confidential data.*<br><br>*The data underlying the results presented in the study are available from (include the name of the third party*	All relevant data are within the manuscript.

and contact information or URL).<br>• This text is appropriate if the data are owned by a third party and authors do not have permission to share the data.<br><br>* typeset	
Additional data availability information:	

Powered by Editorial Manager® and ProduXion Manager® from Aries Systems Corporation

Title Page

Corporate Governance, Financial Performance and Economic Policy Uncertainty: Evidence From Emerging Asian Economies

Chaoyang Zhang[1*]

[1] School of Accountancy, Henan Institute of Economics and Trade, Zhengzhou 450046, Henan, China

*** Corresponding author**

E-mail: vhikyh@qq.com (CZ)

Author's contribution: All authors contributed equally to this work

Competing Interest: Not Applicable

Funding statement: This study did not require any funding

Acknowledgment : Not Applicable

Corporate Governance, Financial Performance and Economic Policy Uncertainty: Evidence From Emerging Asian Economies

Abstract

The objective of this study is to examine the impact of corporate governance and economic policy uncertainty on financial performance in non-financial firms in three Asian emerging economies, Pakistan, China, and Malaysia. The study analyzed 400 non-financial firms listed on the stock markets of these countries for a period of 10 years (2012-2021). Data on corporate governance, financial performance, and CSR scores were obtained from DataStream. The study used regression analysis and the generalized method of moments (GMM) for its robustness analysis. Our findings show that all attributes of the corporate governance practices have significant positive impact on return on asset except existence of audit board committee with respect to all selected economies. Moreover, corporate governance practices have a significant positive relationship with return on equity. However, in the case of EPS all attributes are significant positive relationship except board size with earning per share. The economic policy uncertainty has significant moderating impact between corporate governance practices and financial performance of organizations belongs to Asian economies. This study advocated the implications for the government and policy makers to improve the corporate governance practices especially during the period of high economic uncertainty.

Keywords: Corporate Governance, Financial Performance, Economic Policy Uncertainty, And Emerging Economies

1. Introduction

The financial performance of the corporation plays an important role in the development of the countries with rise to income level of the shareholders because a company's financial health and viability can be inferred from financial performance parameter. These parameters assist stakeholders in determining if the business is making enough money to pay its debts and cover its expenses. Shareholders and prospective investors keep a careful eye on a company's financial results. Positive financial outcomes increase stock price and investor confidence, which makes it

simpler for the business to raise funds through equity financing. Moreover, financial performance information is used by creditors and lenders to assess a company's creditworthiness. A corporation gets financing when necessary and obtains advantageous borrowing terms with the aid of a solid financial history. However, the financial performance of corporations has a broader economic impact. It contributes to overall economic growth by creating jobs, generating tax revenue, and fostering innovation.

Similarly, corporate financial performance is remaining a problem because it affected by many factors such as social and economic factors. However, it is interesting to investigate the financial performance is affected by the corporate governance practices and economic policy uincertainity in this study. The strong corporate governance practices are linked to the success and long-term sustainability of the corporations and provides a correct direction to the business operations in the country. Therefore, smooth corporate governance practices should be necessary for each firm to develop with respect to time. Moreover, accountability and openness are stressed in strong corporate governance standards when it comes to financial reporting and decision-making. A company's stakeholders are more likely to have faith and confidence in it when it provides them with accurate and transparent financial information. This can then draw in investors and possibly cut the cost of capital, which would result in better financial performance. However, investors and stakeholders are increasingly taking into account a company's risk associated with the economic policies when assessing its financial success. A company's reputation and attractiveness to socially conscious investors can be improved by having sound corporate governance policies that address economic policy uncertainty.

Most of the pertinent research endeavors have delved into the primary and frequently prevailing facets of corporate governance, including board size, board characteristics, and other strategic practices that significantly influence corporate financial performance (Hendry & Kiel, 2004). In this way, firms get more profitability to pick the highly efficient mode of management and considering efficient corporate governance practices Jo and Harjoto (2011). There is rich potential available in resolving the doubt regarding the corporate governance mechanism's broader role that could be beneficial for the firms, although it is still unresolved due to wide range of the significance with respect to time domain changes and difference in culture of each economy (Allen, Carletti, & Marquez, 2007; Bai, Ullah, Arif, Erfanian, & Urooge, 2023).

Similarly, the economic policy uncertainty has wide range of the effect on the economy as well as on the financial performance of the organization (Prior, Surroca, & Tribó, 2008; Szczepankiewicz, Błażyńska, Zaleska, Ullah, & Loopesko, 2022). Moreover, corporate governance practices with the economic policy uncertainty have different consequences on the financial performance. Therefore, the investigation about corporate governance practice relationship with financial performance in respect to Asian emerging economy create the research gap for the further investigation. Moreover, the economic policy uncertainty role as moderator to act as key moderating driver between the corporate governance practices and financial performance is another gap for investigation in the study with different motives. Moreover, the previous studies have the data limitation on the economic policy uncertainty, but this study select the gap for investigation with new insight with inclusion of wide range of dataset.

This study encompasses three primary objectives. First, it aims to scrutinize the relationship between various attributes of corporate governance and corporate financial performance by utilizing diverse measures such as return on assets (ROA), return on equity (ROE), and earnings per share (EPS). The second objective involves an examination of the relationship between corporate governance and corporate financial performance within various country contexts, particularly in Asian emerging economies. The third objective is to assess the moderating influence of economic policy uncertainty on the relationship between corporate governance and corporate financial performance.

This study makes significant new contributions in multiple dimensions, effectively filling gaps left by previous researchers. First and foremost, it broadens the scope by encompassing 400 top-listed corporations from three distinct stock exchanges, resulting in a much larger sample size. Previous research, such as Boachie (2023); Pamungkas (2023) used small samples from a single country. Third, whereas previous research, such as Rusmita, An-Nafis, Ramadhani, and Irfan (2023); Singh and Rastogi (2023) frequently focused on short time periods, this study broadens its scope to include a 10-year sample span. Fourth, unlike previous studies that focused on a single nation as their sample, such as Boachie (2023); Pamungkas (2023), this research broadens the reach to include enterprises from three different Asian countries. Fifth, this study distinguishes itself by applying advanced econometric methods, such as composite effects that take into account all listed enterprises in all selected nations. Furthermore, in contrast to previous investigations, such as Singh and Rastogi (2023), which relied on simpler methodologies, separate country-

specific effects were studied using dummy analysis. Finally, to obtain a more accurate result, economic policy uncertainty is used as moderator that has the positive moderating effect.

The rest of the article is organized as follows: Section 2 provides an overview of research theoretical underpinnings, giving many testable hypotheses aimed at understanding variable interactions. The third section describes the conceptual framework, research design, and methodology used. Section 4 presents the study's findings, while Section 5 includes the conclusion, future research suggestions, limitations, practical implications, and policy implications in figure 1.

2. Literature review

2.1 Theoretical background

Every research study is grounded in foundational theories. This study draws upon agency theory, stewardship theory, stakeholder theory, and resource dependence theory as its theoretical underpinning's theory (Athar, Chughtai, & Rashid, 2023; Gwala & Mashau, 2023; Steinfeld, 2023). Corporate governance, according to these foundational theories, is critical in resolving conflicts between management and shareholders, aligning management objectives with the interests of investors, and maximizing corporate financial (Bai et al., 2023; D'Augusta, Grossetti, & Imperatore, 2023). These theories also advocate for management to act in the best interests of both shareholders and stakeholders.

This study is also justified by stakeholder theory, which emphasizes the prioritizing of stakeholders through the protection of their rights and the production of long-term value through good corporate governance processes and CSR disclosures. This study is supported by agency theory, which provides guidance on the separation of ownership and control, which supports solid governance practices in the country and improves an organization's financial performance (Jensen & Meckling, 1976).

Numerous studies have explored the relationship between corporate governance and corporate financial performance and have produced a body of evidence that supports the notion that effective corporate governance practices significantly influence a firm's financial and operational performance such as (Saderanta, 2022) define the same relationship and concluded the positive relationship between them. Furthermore, (Bandari, 2023) investigates the effect of

corporate governance on firm performance, notably in the context of the COVID-19 epidemic. Their findings show that companies with strong corporate governance measures, including board independence and CEO duality, fared better during the pandemics and maintained their financial performance. Notably, good corporate governance emphasizes the significance of risk management. Firms that have well-established risk management systems are better positioned to recognize, assess, and mitigate a wide range of risks, including financial, operational, legal, and reputational concerns. This astute risk management strategy aims to avoid potential financial losses and protect the company's assets, consequently strengthening its financial performance (Jones Khosla & Gomes, 2023).

2.2 Governance practices and corporate financial performance

The corporate governance practices and the financial performance of the firms are widely discussed in many literatures. The corporate governance practices vary across the country depending on the environment and culture of the economy. In many countries there are family-owned firms, but other ones may the public owned firms. According to Halim, Mustika, Sari, Anugerah, and Mohd-Sanusi (2017), the efficient corporate governance practices increase the corporate performance by reducing the risk factors, increasing the decision efficiency and the helped in decision making that improve the performance directly. The audit committee and the board characteristics that followed the code of the corporate governance improve the financial performance in term increasing return and profitability (Wang, Su, Wang, & Chen, 2017).

H. J. Huang, Habib, Sun, Liu, and Guo (2021) also found the significant positive relationship between corporate governance practice and the performance and concluded the positive effects of the independent board on performance in China. Moreover, an efficient board improve the financial performance by taking timely decisions, timely solving problems and taking opportunities by protection and decision making (Gold & Taib, 2023).

Riyadi, Arif, Halik, and Ariestya (2023) investigated the positive relationship between board size and performance and concluded that the larger board has the significant sign for organization promotion due to effective decision making and brainstorming that also reduce the agency problems. Similarly, Almashhadani and Almashhadani (2023) found the negative consequences of the larger board on the performance due to increase in corporation expenses that

leads to decrease the profitability. However, Khanal (2023) concluded the positive relationship between larger board and performance.

Joecks, Pull, and Scharfenkamp (2023) found the positive relationship between board diligence attendance and performance and also concluded that the board attendance of the directors in meeting increase the brainstorming and thinking power of the board for taking timely decisions. Similarly, Govindan, Karaman, Uyar, and Kilic (2023) investigated the positive effects of the presents of the board of directors on profitability due to management of the top level decisions.

Khan, Afeef, Ilyas, and Jan (2023) investigated that the audit committee attract the foreign investor due to increase in the investors' confidence on the firms due to mitigation of the risk of frauds. Alabdullah (2023) explained the positive consequences of the audit committee on profitability due to risk reduction and removing weakness of internal control. Moreover, Hanjani and Kusumadewi (2023) define the positive relationship between audit committee and performance. Alodat, Al Amosh, Khatib, and Mansour (2023) investigated that financial performance is also positively affected by the expertise and independence of the audit committee, attributed to their wealth of experience and impartial perspectives. Based on the theoretical and empirical evidence, we developed the following hypothesis,

H1: Board size has a significant positive impact on performance.

H2**:** Board attendance has a significant positive impact on performance.

H3**:** CSR committee existence has a significant positive impact on financial performance.

H4*:* Audit board committee has a significant positive impact on performance.

H5: Audit committee independence has a significant positive impact on financial performance.

H6: Audit committee expertise has a significant positive impact on financial performance.

2.3 Moderating role of economic policy uncertainty

Economic policy uncertainty affects different countries in various ways, depending on their economic structures, political systems, and exposure to global markets. While some nations may experience increased caution and reduced investment during periods of uncertainty, others may respond with adaptive strategies, innovation, and diversification, underscoring the complexity of its global impact.

Economic policy uncertainty exerts a notable influence on corporate governance practices, as evidenced in recent literature. Scholars have increasingly examined how fluctuations in economic policy uncertainty impact corporate decision-making, risk management, and overall governance strategies. High levels of economic policy uncertainty often led companies to adopt a more cautious approach, potentially affecting their investment decisions, dividend policies, and executive compensation structures (Qian, 2023). A climate of uncertainty also stimulates a stronger emphasis on risk management within organizations, as companies seek to navigate turbulent economic landscapes and safeguard their financial interests (Settembre-Blundo, González-Sánchez, Medina-Salgado, & García-Muiña, 2021). Moreover, (Zhang, Chen, & Hu, 2023) suggests that companies facing elevated economic policy uncertainty may pay greater attention to corporate governance mechanisms as a means to mitigate potential risks and maintain investor confidence. These findings underscore the complex interplay between economic policy uncertainty and corporate governance practices, offering valuable insights into how external economic factors can shape internal governance dynamics and, ultimately, impact firm performance and stakeholder interests.

Various literature has extensively investigated the impact of economic policy uncertainty on corporate financial performance. Studies have shown that higher levels of economic policy uncertainty can lead to decreased investment, reduced firm valuations, and increased risk premiums, ultimately exerting a negative influence on financial performance (Baker, Davis, & Levy, 2022). Firms in uncertain economic environments often become more cautious in their decision-making, leading to delayed investments and a focus on liquidity management to mitigate potential risks (Liu, Li, Liu, & Lee, 2021). Moreover, economic policy uncertainty can affect firms' access to capital, as lenders and investors may become more risk-averse, leading to higher financing costs (Long, Zhang, & Hao, 2022). However, it is essential to note that the specific effects of economic policy uncertainty can vary across industries, regions, and firm sizes, reflecting the nuanced relationship between economic policy dynamics and corporate financial performance. Furthermore, some firms may exhibit adaptability and innovation in response to uncertainty, which can positively influence their financial outcomes, emphasizing the multidimensional nature of this relationship (H. Huang, Liu, & Yang, 2021). Hence, on the ground of previous literature discussion, we assume the positive moderating influence of economic policy uncertainty. On the grounds of discussion, we developed the following hypothesis.

H7: Economic policy uncertainty has significant positive moderating impact between corporate governance attributes and financial performance.

3. Research Design

3.1. Data collection and sample size

To meet our objectives of the study, this study used the 400 firms' data from three different economies belongs to Asian region from 2012 to 2021 on annually frequency. Pakistan, China and Malaysia three countries from the Asian region selected the sample because recently these three countries that has the major portfolio and the population effected by the climate change such as water flood caused the loss in Pakistan and India recently due to the climate change. Pakistan and India both are most polluted country as air pollution index are high of these countries, while China has the major trading partners and want sustainable environment for neighborhood economies by regional cooperations.

Moreover, an agreement between these economies conducted in Shanghai Cooperation Organization (SCO) meeting recently for the development of the poor climate managed countries. The Shanghai Cooperation Organization (SCO) countries made a plan for the reduction of the carbon emission through the member economies and the economic policy frequently changed according to that situation depends on the regions. The time period chosen for analysis (2012-2021) is significant due to the changes made by certain economies during this period with regards certain policies and their financials. Non-financial companies were selected for analysis due to the distinct social, cultural, and regulatory settings that differ from those observed in the financial sector, where similar cultural and regulatory settings are commonplace.

There are two sources of data collection. First, the data related to corporate governance, Economic policy uncertainity, firm age, firm size is collected from the DataStream database. Second, the data related to GDP data for these years are extracted from the world bank data portal. The sample size from each economy is given below in table 1. The sampling techniques used in the study is convenience sampling which depends on the availability of the data of these firms in the respected economies. Table 3.1 displays the sample size for each economy. The sample comprises the higher market capitalized companies from each country selected through random sampling techniques of the probability sampling. These companies represent a significant contribution to the total market capitalization of their respective countries.

Table 1: Number of the firms taking as a sample

Name of the Country	Non-Financial firms Sample
Pakistan	150
China	127
Malaysia	123

Figure 1: Conceptual framework of study

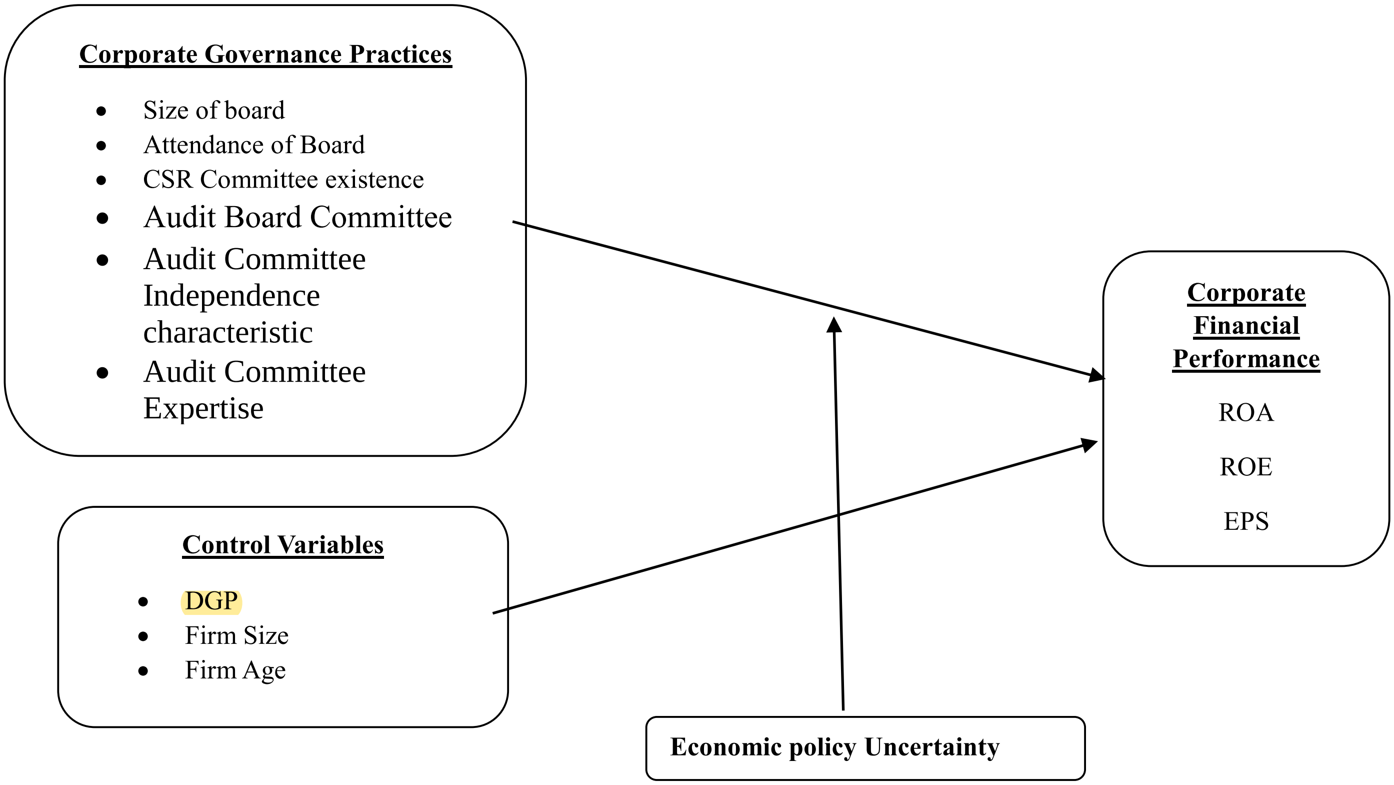

3.2 Variables Measurement

3.2.1 Dependent Variable

The key dependent variable under consideration in this study is financial performance, which is measured using several measures. Return on assets (ROA), return on equity (ROE), and earnings per share (EPS) are examples of these indicators. Among these indicators, return on assets (ROA) is well-known in finance and is frequently used to assess business financial performance. ROA is calculated by dividing a company's net profit by its total assets, and it serves as an indicator of how well a company leverages its assets to maximize output. The return on asset is consider most important for measuring the financial performance (Lakonishok, Shleifer, Vishny, Hart, & Perry, 1992). Furthermore, return on equity (ROE) is a popular metric for assessing business financial performance. It is calculated by dividing net income by total shareholders' equity, and it reflects the effective use of owner's equity to achieve profitability. The utilization of the equity is essential for generating the profitability (Almajali, Alamro, & Al-Soub, 2012). Furthermore, earnings per share (EPS) is a third indicator used to assess financial success. Profitability is a commonly acknowledged metric for evaluating business financial performance (Tabassum, Kaleem, & Nazir, 2013).

3.2.2 Independent variable

This study's independent variable is corporate governance practices, which have been evaluated based on a variety of criteria, including board and auditor characteristics. Notably, corporate governance practices taken into account in this study include board size, board attendance, and the inclusion of CSR suitability committees on the board. Furthermore, auditor-related qualities include the existence of an audit board committee, auditor independence, and audit committee expertise. Furthermore, the total number of board directors within the board composition is used to establish board size. It is crucial to remember that the criteria for maximum and minimum board members change among nations due to differences in corporate governance regulations (Cheung & Chan, 2004). Board attendance measured by the presence of the board member in meeting (Adams & Ferreira, 2012) and board CSR suitability committees is measured through the dummy, if existed then denoted by 1, if not the 0. The audit board committee is also measured through dummy, if existed then denoted by 1, if not the 0. Audit committee expertise

score is used for measuring the auditor committee expertise (Cohen, Hoitash, Krishnamoorthy, & Wright, 2014).

3.2.3 Control variables

Control factors such as GDP, firm size (FSIZ), and firm age (AGE) are included in this study. Total assets are used to determine business size since they are a key factor influencing firm performance. GDP, which measures economic output inside a certain country, has an impact on the financial success of enterprises operating in that country. Meanwhile, firm age is measured in years since the date of incorporation in the stock market, providing insight into the maturity of enterprises relative to total market capitalization.

3.2.4 Moderator

The moderator variable is the economic policy uncertainty in this study. Economic policy uncertainty creates an index based on three major components to measure policy-related economic uncertainty (Baker, Bloom, & Davis, 2016). The first, and most adjustable, component estimates the amount to which policy-related economic uncertainty is covered in the media. The second component of ranking is based on information compiled by the Congressional Budget Office (CBO), which compiles lists of temporary provisions in the federal tax code. The policy-related uncertainty index's third component is drawn from the Federal Reserve Bank of Philadelphia's Survey of Professional Forecasters.

3.3 Econometric Model and Method of estimation

This study empirically examined the impact of corporate governance on firm performance within listed companies operating in three emerging economies within a specific region over a 10-year period. Drawing on pertinent literature, this research employed regression analysis techniques.

$$\mathbf{FP_{it}=\alpha_0+\beta_1(CG)_{it}+\beta_2(EPU)+\varepsilon_{it}}$$

$$\mathbf{FP_{it}=\alpha_0+\beta_1(CG)_{it}+\beta_2(CG)*EPU_{it}+\varepsilon_{it}}$$

Regression analysis serves as a widely adopted approach for assessing the connection between financial variables. It entails the estimation of the association between a dependent variable and one or multiple independent variables. In the realm of finance, the dependent variable frequently represents a measure of financial performance, such as return on assets or stock price, while the independent variables encompass factors that could potentially impact the dependent variable, including interest rates, inflation, and firm-specific variables like size or leverage. In finance and economics, regression analysis is a prevalent and valuable statistical tool employed to investigate and understand the relationships between variables. Chen, Wang, Albitar, and Huang (2021) used the regression method to analyze the risk in context of China. Moreover, Apergis, Ewing, and Payne (2021) investigate the volatility relationship among oil prices, and stock market using the regression analysis method

Moreover, the regression analysis has a weakness in terms of the simultaneously effect found between the endogenous and exogenous variables. To remove the simultaneously effect and problem of the autocorrelation (if any) between endogenous and exogenous variables, there should be the use of the generalized method of movement (GMM) that is useful in econometrics. For our findings robustness we used the GMM method for real impact. Therefore, in instrumental variable regression models in econometrics, weak instruments can result in skewed parameter estimations. When instrumental variable approaches are required, GMM is an important tool since it offers a mechanism to test for and address the issue of weak instruments.

4. Results and Findings

4.1 Descriptive statistics

Table 2: Descriptive statistics of the samples

	**Mean**	**Maximum**	**Minimum**	**Std. Dev.**	**Observations**
**ROA**	0.33	616.20	-2.10	11.18	4000
**ROE**	5.84	16171.28	-157.95	291.48	4000
**EPS**	1.13	193.81	-12.37	6.70	4000
**EPU**	139.13	199.22	0.00	30.70	4000

**ABC**	0.85	1.00	0.00	0.36	4000
**AC**	51.47	100.00	0.00	32.33	4000
**ACI**	67.00	100.00	0.00	37.16	4000
**AGE**	15.99	120.00	1.00	14.97	4000
**BA**	0.45	1.00	0.00	0.50	4000
**BS**	11.00	19.00	7.00	7.09	4000
**CSC**	0.66	1.00	0.00	0.47	4000

In Table 2 above, the selected firms exhibit an average return on assets of 32.29%, indicating their ability to generate, on average, a 32.29% return on their assets. Moreover, the average return on equity for these firms is notably five times higher than their average ROA, underscoring a favorable return on shareholders' equity. The mean value of earnings per share stands at 1.126, indicating a consistent positive trend in firm earnings over the past decade. Furthermore, Economic policy uncertainty has a mean value of 139.13, with a range from 0 to 199 among the selected firms in Asian economies. Approximately 51% of firms feature an audit committee, and the firms, on average, have been in operation for 16 years since their incorporation. Furthermore, about 44% of firms have board attendance at board meetings, and the average board size comprises 11 members. Among the Asian economy data, 66% of firms have a CSR committee.

Table 3 indicates that there are no correlations exceeding the 0.80 threshold between variables. When the correlation between variables exceeds 0.80, it may hinder regression analysis. In our case, the absence of such strong correlations allows for a suitable regression analysis. While there is a correlation between ROA and ROE, both of which are dependent variables, since only one measure of financial performance is employed, it does not significantly impact the overall regression results.

4.2 Results and Findings

The regression analysis in Tables 4 sheds light on the impact of corporate governance on proxies for company financial performance, specifically in the context of Pakistan, as represented by the country dummy. The R-squared values of 0.7410, 0.7982, and 0.4210 represent significant

variations in the dependent variables ROA, ROE, and EPS due to the explanatory variables, which include board-related and audit-related characteristics of corporate governance mechanism, and are statistically significant at the 1%, 5%, and 10% levels of significance, respectively. Furthermore, the positive F-statistics highlight the model's appropriateness and fitness in explaining the observed outcomes.

The presence of the audit board committee (ABC) does not have a statistically significant impact on performance, as indicated by non-compliance with acceptable probability criteria, according to the study of ROA results. Our null hypothesis is confirmed in this finding. However, at the 5% level of significance, the presence and independence of the audit committee have a significant and favorable effect on performance. This means that a one-unit increase in the audit committee's existence and independence corresponds to a 0.001 and 0.002 increase in ROA, respectively. Furthermore, all other components of corporate governance have a significant and positive influence, supporting the view that good governance procedures improve business financial performance. Furthermore, corporate governance practices across all countries appear to have a significant positive impact on improved financial performance, with the exception of China, where they appear to have a significant negative impact, possibly influenced by cultural values or other factors.

Table 3: Correlation Matrix

	ROA	ROE	EPS	EPU	ABC	AC	ACI	AGE	BA	BS	GDP	FS
**ROA**	1											
**ROE**	1	1										
**EPS**	0	0	1									
**EPU**	-0.03	-0.03	0.08	1								
**ABC**	-0.04	-0.04	0.06	0.59	1							
**AC**	-0.03	-0.03	-0.03	0.43	0.64	1						
**ACI**	-0.03	-0.03	-0.06	0.47	0.66	0.55	1					
**AGE**	0	-0.01	-0.02	-0.05	-0.04	-0.02	-0.05	1				
**BA**	-0.01	-0.01	0.02	0.17	0.35	0.35	0.44	-0.01	1			
**BS**	0	0	-0.01	0	0.02	-0.02	0.02	-0.01	0.03	1		
**CSC**	-0.03	-0.03	0.01	0.65	0.54	0.42	0.47	-0.07	0.24	-0.06		
**GDP**	-0.01	-0.01	0.03	0.18	0.21	0.12	0.11	-0.36	0.08	-0.08	1	
**FS**	0	0	0.04	0.14	0.01	0.01	0	0.02	-0.03	0	0.02	1

Author source

In the investigation of ROE results, it is worth noting that all corporate governance attributes, as well as control variables, have a positive and statistically significant relationship with the firm's financial success, highlighting the critical role of effective governance in a firm's development. This means that a one-unit rise in corporate governance variables correlates to a one-unit increase in ROE based on the value. However, China has a large negative impact, which may be related to cultural values or other contributing factors.

The examination of EPS findings shows that, with the exception of board size and board attendance, all attributes have a statistically significant positive influence on performance. Notably, there is a considerable negative influence on board attendance, implying that a large number of participants may disturb the board's decision-making process and squander valuable time and resources. Furthermore, Malaysia is the only country with a positive effect, while other Asian economies show no distinct benefits. Finally, our findings highlight the important influence of corporate governance policies on business financial success. For more accuracy of results by removing the simultaneously effect we used the generalized methods of movement (GMM) by taking the lag difference techniques in table 4.

Table 4: Results of Regressions

	**ROA**		**ROE**		**EPS**	
**Variable**	Coefficient	Prob.	Coefficient	Prob.	Coefficient	Prob.
**ABC**	0.677	0.134	39.541	0.076	3.871	0.000
**AC**	0.001	0.017	0.001	0.097	0.020	0.000
**ACI**	0.002	0.018	0.070	0.009	0.033	0.000
**AGE**	0.007	0.090	0.178	0.006	0.009	0.155
**BA**	0.167	0.001	2.441	0.076	-0.868	0.000
**BS**	0.000	0.834	0.000	0.025	0.000	0.302
**CSC**	0.297	0.083	2.013	0.055	-0.934	0.000
**EPU**	0.006	0.025	0.097	0.035	0.021	0.000
**FS**	-0.001	0.008	0.013	0.044	0.008	0.071
**GDP**	0.000	0.667	0.000	0.017	0.000	0.919

**China**	-0.241	0.0263	-7.218	0.076	0.117	0.686
**Malaysia**	2.059	0.0224	29.746	0.069	0.351	0.559
**C**	1.775	0.0003	42.572	0.007	0.358	0.307
**R-squared Value**	0.20410		0.0982		0.4210	
**Adjusted R**	0.20301		0.0871		0.4014	
**Probability**	0.0000		0.0000		0.0000	
**Durbin-Watson**	1.0104		1.1701		1.1214	

Note. Findings are presented with respect to Pakistan as country dummy.

4.3 Robustness analysis: Generalized Method of Movement (GMM)

The regression analysis has some weakness in the econometrics analysis due to the current conditions. In the current environment of the business the variables are classified as structural that shows the dual causal relationship between each other that creates a critical point on the validity of the regression analysis. The simultaneously structuring of the different factors motivate the use of the simultaneous equation modeling in the endogenous variables. To diagnose the real impact of the variables on the endogenous variables, we used the generalized method of movement (GMM) with the help of the instruments. In this study, we used the difference GMM to investigate the real impact of the exogenous variable on the endogenous. The motivation behind this method is the simultaneous effect of the variables such as ROA can increase the CSR disclosure and CSR disclosure can enhance the reputations that leads to rise in the profitability and vice versa. The GMM methods results shows that the J-Statistics value is favorable, and probability of J-Statistics is significant at 1% level of the significance indicating the excellent estimations of the models and also shows the reliable results for interpretation. Moreover, GMM findings in table 5 show that the lag value of ROA and EPS affect the current value with different ratios and contributed positively.

Table 5: Robust analysis through GMM method

	**ROA**		**ROE**		**EPS**	
**Variable**	Coefficient	Prob.	Coefficient	Prob.	Coefficient	Prob.

**ROA (-1)**	0.011	0.001	-	-	-	-
**ROE (-1)**	-	-	0.001	0.000	-	-
**EPS (-1)**	-	-	-	-	0.001	0.058
**ABC**	0.423	0.093	37.871	0.057	8.921	0.000
**AC**	0.098	0.000	0.012	0.000	0.020	0.000
**ACI**	0.018	0.000	0.180	0.000	0.087	0.000
**AGE**	0.170	0.000	0.179	0.000	0.019	0.000
**BA**	0.231	0.000	2.331	0.000	-0.976	0.000
**BS**	1.520	0.000	2.191	0.000	3.171	0.000
**CSC**	0.176	0.000	2.130	0.000	-0.934	0.000
**EPU**	0.012	0.000	0.097	0.000	0.089	0.000
**FS**	-0.001	0.000	0.019	0.000	0.019	0.000
**GDP**	2.721	0.000	3.912	0.010	4.752	0.761
**China**	-0.241	0.026	-9.918	0.000	0.117	0.686
**Malaysia**	2.059	0.022	32.746	0.000	0.351	0.559
**J-Statistics**	0.909		0.721		0.911	
**Prob(J-statistics)**	0.005		0.001		0.002	

Note. Findings are presented with respect to Pakistan as country dummy.

Our results show that all the results improved with greater frequency and intensity as compared with the regression analysis with the removal of the simultaneous effects. In all models, GMM estimations show that the intensity of the effects improved such as GDP, FS and ENV impact increased on the corporate financial performance. In our results the direction of the significance is same as compared with the regression analysis, so we rely on the findings of the GMM methods because the problem of the autocorrelation that existed in the regression analysis are removed. In the ROA results, the audit board committee (ABC) has no significant impact on performance, as it did not follow the accepted criteria of probability value in the regression results, by employing the GMM methods it becomes significant and positive impact on ROA. Finally, we can conclude

that GMM methods improve the findings of the regression analysis by removing the dual causal relationship and represents the real impact only.

4.4. Effect of the moderator variable

The R-squared values for our models, namely return on assets (ROA), return on equity (ROE), and earnings per share (EPS), were calculated as 0.449, 0.439, and 0.129, respectively, when the moderating effects of economic policy uncertainty were examined, as shown in Table 6. These statistics show that the explanatory variable of corporate governance has a considerable influence on the dependent variables, indicating that the model is fit for purpose. Furthermore, all calculated models had positive F-statistic values, confirming the accuracy of the estimates. The Durbin-Watson values were likewise favorable, close to 2.00, indicating that the calculated models had favorable autocorrelation values.

In Table 6, it was found that the results of Economic policy uncertainty were negative and significant moderating impact between corporate governance practices and corporate financial performance. This means that countries with higher economic policy uncertainty tend to have lower profitability in terms of ROA, ROE and EPS. Moreover, the findings show that economic policy uncertainty has a notable and statistically significant moderating effect on the connection between the audit committee and firm financial performance. The negative indication indicates that in the context of significant economic policy uncertainty, the audit committee's influence on financial performance is less beneficial. This means that the audit committee's positive impact on financial performance may be diminished during periods of economic insecurity and unpredictability. Firms with competent audit committees are more likely to ensure openness, risk management, and accountability. When economic policy uncertainty looms large, organizations may have heightened problems in managing economic conditions and maintaining financial performance, even if they have a strong audit committee in place.

Similarly, the study also finds that economic policy uncertainty has a significant negative moderating influence on the connection between audit committee independence and firm financial performance. This shows that when economic policy uncertainty is high, the favorable impact of an independent audit committee on financial performance is lessened. These findings highlight the

necessity of taking into account external economic conditions when evaluating the influence of corporate governance policies on financial outcomes.

However, the study finds that economic policy uncertainty has a significant negative moderating influence on the connection between board size and firm financial performance. This means that the positive benefit of a larger board on financial performance lessens during periods of high economic policy uncertainty. These findings emphasize the dynamic interaction of external economic variables and corporate governance dynamics in affecting financial outcomes.

The study finds that economic policy uncertainty has a significant negative moderating influence on the relationship between board attendance and business financial success. This shows that when economic policy uncertainty is high, the positive impact of board participation on financial performance diminishes. These findings highlight the contextual aspect of the relationship between corporate governance standards and financial outcomes, especially in the face of economic policy uncertainty. The effect of each economy is different due to the change of culture and other norms within the country.

Table 6: Results of Moderation Economic Policy Uncertainty

	**ROA**		**ROE**		**EPS**	
**Variable**	Coefficient	Prob.	Coefficient	Prob.	Coefficient	Prob.
**ABC*EPU**	-0.008	0.000	-0.231	0.006	-0.013	0.001
**AC*EPU**	-0.013	0.007	-0.000	0.004	-0.001	0.007
**ACI*EPU**	-0.135	0.002	-0.006	0.006	-0.001	0.000
**BA*EPU**	-0.001	0.000	-0.178	0.007	-0.004	0.001
**BS*EPU**	-2.132	0.000	-2.873	0.876	-0.006	0.655
**China**	-0.037	0.000	-1.164	0.001	-0.198	0.342
**Malaysia**	-0.041	0.000	-1.089	0.005	-0.766	0.000
**R-Value**	0.449		0.431		0.129	
**Adjusted R**	0.416		0.421		0.119	
**Probability**	0.000		0.000		0.000	

**Durbin-Watson**	1.991		2.001		2.101

5. Discussion

The audit board committee (ABC) has not affected the corporate financial performance due to the cultural and policy differences across the countries around the globe. Nonetheless, the independence of the audit committee demonstrates a noteworthy and positive impact on performance, aligning with prior research findings (Al-Matari, Al-Swidi, FADZİL, & Al-Matari, 2012). Furthermore, all other dimensions of corporate governance exhibit significant and positive effects, underscoring the potential of sound governance to enhance financial performance. Notably, when examining the impact across various countries, it becomes evident that only China exhibits a significant negative effect of corporate governance on firm performance, whereas all other Asian economies display a positive effect consistent with the composite effect observed in the overall analysis. These findings hold relevance for companies operating in diverse countries as they contemplate efforts to enhance their corporate governance practices. Although good governance is commonly linked to enhanced financial performance, it is crucial to consider the unique country-specific context and potential disparities in the influence of distinct governance practices (Filatotchev & Wright, 2017).

In this regard, our findings align with previous literature, reinforcing the idea that effective corporate governance can indeed yield a positive impact on corporate financial performance (Van Essen, Engelen, & Carney, 2013). Notably, several key attributes of corporate governance have been linked to enhanced performance, including board independence, corroborating findings similar to those of Sanda (2011), who established that companies with independent boards tend to exhibit superior financial performance due to the objective oversight provided by independent directors, which helps mitigate conflicts of interest. Overall, our results are consistent with the broader body of literature suggesting that strong corporate governance practices play a pivotal role in bolstering corporate financial performance, and firms that prioritize these practices are poised for long-term success (Puni & Anlesinya, 2020). In our findings, the significant moderating impact of Economic Policy Uncertainty on the relationship between corporate governance and financial

performance is in line with the observations of Gelb and Strawser (2001), who found that companies facing high levels of economic uncertainty experienced decreased stock returns.

Moreover. Economic policy uncertainty can exert a detrimental influence on performance across various domains, including financial markets and business operations. High levels of economic policy uncertainty often led to increased caution and risk aversion among investors and firms. Businesses may postpone investments, delay strategic decisions, and prioritize liquidity management to hedge against potential adverse economic developments. Uncertainty can also contribute to elevated financing costs, reducing firms' access to capital and potentially impacting their ability to grow and innovate. In financial markets, heightened economic policy uncertainty can lead to increased market volatility and reduced investor confidence, resulting in decreased stock prices and returns. In sum, economic policy uncertainty can cast a shadow of apprehension, impacting performance by hindering investment, increasing costs, and generating a climate of unpredictability that can undermine both corporate and financial sector performance.

6. Conclusion

From our study we concluded that all attributes of corporate governance increase the financial performance and economic policy uncertainty provide the significant negative moderating impact between them. This study makes significant new contributions in multiple dimensions, effectively filling gaps left by previous researchers. First and foremost, it broadens the scope by encompassing 400 top-listed corporations from three distinct stock exchanges, resulting in a much larger sample size. Previous research, such as Boachie (2023); Pamungkas (2023) used small samples from a single country. Third, whereas previous research, such as Rusmita et al. (2023); Singh and Rastogi (2023) frequently focused on short time periods, this study broadens its scope to include a 10-year sample span. Fourth, unlike previous studies that focused on a single nation as their sample, such as Boachie (2023); Pamungkas (2023), this research broadens the reach to include enterprises from three different Asian countries. Fifth, this study distinguishes itself by applying advanced econometric methods, such as composite effects that take into account all listed enterprises in all selected nations. Furthermore, in contrast to previous investigations, such as Singh and Rastogi (2023), which relied on simpler methodologies, separate country-specific effects were studied using dummy analysis. Finally, to obtain a more

accurate result, economic policy uncertainty is used as moderator that has the positive moderating effect.

The study has several policy implications for corporations and policymakers. Corporations should prioritize good corporate governance practices, as they have a significant impact on financial performance. This includes establishing effective board structures, adopting transparent and ethical business practices, and implementing adequate risk management policies. Investors and stakeholders should consider both corporate governance and economic policy uncertainty when making investment decisions. Companies that prioritize both governance and policy uncertainty tend to have better financial performance and are more likely to mitigate risks. Moreover, the study's findings hold significant policy implications for both businesses and governments. They underscore the critical importance of fostering robust corporate governance practices, especially in environments marked by economic policy uncertainty. Businesses that prioritize independence within their boards and transparent governance mechanisms are more likely to weather economic turbulence successfully. For policymakers, this highlights the need for stable and consistent economic policies to reduce uncertainty, promoting investor and business confidence. Ultimately, enhancing corporate governance practices and providing a predictable policy environment can contribute to economic resilience, greater financial stability, and sustainable growth. So, the key takeaway is clear: Strong corporate governance and stable economic policies create a more attractive landscape for business success and economic prosperity.

Research is an ever-evolving process, and not all studies can comprehensively cover every facet of the subject matter. Consequently, this study comes with certain limitations but also paves the way for future investigations. The sample size in this study is limited to 400 firms, presenting an opportunity for future research to expand the dataset, incorporating a wider array of firms and diverse economies from various regions to gain a more comprehensive understanding of the impact of economic policy uncertainty. Additionally, it is crucial to recognize that economic policy uncertainty scores may differ between emerging economies and Asian economies, highlighting the need for future research that focuses on emerging economies and facilitates comparisons between governance practices in developed and emerging economies. Although this study encompasses non-financial firms, future research should delve into financial firms' data. Furthermore, exploring the direct relationship between economic policy uncertainty and corporate financial performance presents another promising avenue for future investigations.

Declarations

Ethics approval and consent to participate

I created the research question, designed the study, and reviewed the literature. They were also critical in data collection and analysis, particularly in the quantitative aspects of the study. All experiments and protocols are done according to the regulations, guidelines and did not require ethical approval. Contributed to the theoretical framework as well as understanding of the underlying financial and economic concepts. They were actively involved in the development of the theoretical background section.

Ethics Approval/Consent to participate

This study adheres to the guidelines of the ethical review process of the associated university and approved by the Institutional Review Committee. The study does not involve humans as a subjects or patients. However, consent was taken from participants before data collection. In compliance with ethical principles, we unequivocally affirm that **informed consent** was obtained from all subjects who participated in this study, as well as from their legal guardian(s). The **informed consent** process was executed with transparency, and participants were provided with comprehensive information about the study. All experiments were performed in accordance with relevant guidelines and regulations described in Declaration of Helsinki.

Consent for publication

Not applicable.

Availability of data and materials

The datasets used and/or analyzed during the current study available from the corresponding author on reasonable request.

Competing interests

The authors confirm no financial and personal relationship or interest.

Funding

There is no funding for the submission of this study.

Authors' contributions

Writing - Original Draft, Methodology, Investigation, Conceptualization. Formal Analysis, Data Curation, Writing - Review & Editing. All are done by the author “**Chaoyang Zhang**”.

Acknowledgements

Not Applicable

References.

Adams, R. B., & Ferreira, D. (2012). Regulatory pressure and bank directors’ incentives to attend board meetings. *International Review of Finance, 12*(2), 227-248.

Al-Matari, Y. A., Al-Swidi, A. K., FADZİL, F. H. B. H., & Al-Matari, E. M. (2012). Board of directors, audit committee characteristics and the performance of Saudi Arabia listed companies. *International Review of Management Marketing, 2*(4), 241-251.

Alabdullah, T. T. Y. (2023). The Role Of Audit Committees In Omani Business Context: Do They Affect The Performance Of Non-Financial Companies. *JOURNAL OF HUMANITIES, SOCIAL SCIENCES & BUSINESS, 2*(4), 643-659.

Allen, F., Carletti, E., & Marquez, R. (2007). Stakeholder capitalism, corporate governance and firm value. *Corporate Governance Frm Value . EFA*, 09-28.

Almajali, A. Y., Alamro, S. A., & Al-Soub, Y. Z. (2012). Factors affecting the financial performance of Jordanian insurance companies listed at Amman Stock Exchange. *Journal of Management research, 4*(2), 266.

Almashhadani, M., & Almashhadani, A. (2023). Corporate Governance Science, Culture and Financial Performance. *International Journal of Business and Management Invention, 11*(2), 55-60.

Alodat, A. Y., Al Amosh, H., Khatib, S. F., & Mansour, M. (2023). Audit committee chair effectiveness and firm performance: The mediating role of sustainability disclosure. *Cogent Business & Management, 10*(1), 2181156.

Apergis, N., Ewing, B. T., & Payne, J. E. (2021). The asymmetric relationship of oil prices and production on drilling rig trajectory. *Resources Policy, 71*, 101990.

Athar, M., Chughtai, S., & Rashid, A. (2023). Corporate governance and bank performance: evidence from banking sector of Pakistan. *Corporate Governance: The International Journal of Business in Society*.

Bai, K., Ullah, F., Arif, M., Erfanian, S., & Urooge, S. (2023). Stakeholder-Centered Corporate Governance and Corporate Sustainable Development: Evidence from CSR Practices in the Top Companies by Market Capitalization at Shanghai Stock Exchange of China. *Sustainability, 15*(4), 2990.

Baker, S. R., Bloom, N., & Davis, S. J. (2016). Measuring economic policy uncertainty. *The quarterly journal of economics, 131*(4), 1593-1636.

Baker, S. R., Davis, S. J., & Levy, J. A. (2022). State-level economic policy uncertainty. *Journal of Monetary Economics, 132*, 81-99.

Bandari, V. (2023). Enterprise Data Security Measures: A Comparative Review of Effectiveness and Risks Across Different Industries and Organization Types. *International Journal of Business Intelligence and Big Data Analytics, 6*(1), 1-11.

Boachie, C. (2023). Corporate governance and financial performance of banks in Ghana: the moderating role of ownership structure. *International Journal of Emerging Markets, 18*(3), 607-632.

Chen, S., Wang, Y., Albitar, K., & Huang, Z. (2021). Does ownership concentration affect corporate environmental responsibility engagement? The mediating role of corporate leverage. *Borsa Istanbul Review, 21*, S13-S24.

Cheung, S. Y., & Chan, B. Y. (2004). Corporate governance in Asia. *Asia Pacific Development Journal, 11*(2), 1-32.

Cohen, J. R., Hoitash, U., Krishnamoorthy, G., & Wright, A. M. (2014). The effect of audit committee industry expertise on monitoring the financial reporting process. *The Accounting Review, 89*(1), 243-273.

D'Augusta, C., Grossetti, F., & Imperatore, C. (2023). Environmental awareness and shareholder proposals: the case of the Deepwater Horizon oil spill disaster. *Corporate Governance: The International Journal of Business in Society*.

Filatotchev, I., & Wright, M. (2017). Methodological issues in governance research: An editor's perspective. *Corporate Governance: An International Review, 25*(6), 454-460.

Gelb, D. S., & Strawser, J. A. (2001). Corporate social responsibility and financial disclosures: An alternative explanation for increased disclosure. *Journal of business ethics, 33*, 1-13.

Gold, N. O., & Taib, F. M. (2023). Corporate governance and extent of corporate sustainability practice: the role of investor activism. *Social Responsibility Journal, 19*(1), 184-210.

Govindan, K., Karaman, A. S., Uyar, A., & Kilic, M. (2023). Board structure and financial performance in the logistics sector: Do contingencies matter? *Logistics and Transportation Review, 176*, 103187.

Gwala, R. S., & Mashau, P. (2023). Tracing the Evolution of Agency Theory in Corporate Governance. In *Governance as a Catalyst for Public Sector Sustainability* (pp. 260-285): IGI Global.

Halim, E. H., Mustika, G., Sari, R. N., Anugerah, R., & Mohd-Sanusi, Z. (2017). Corporate governance practices and financial performance: The mediating effect of risk management committee at manufacturing firms. *Journal of International Studies, 10*(4).

Hanjani, A., & Kusumadewi, R. K. A. (2023). Environmental performance and financial performance: Empirical evidence from Indonesian companies. *Corporate Social Responsibility and Environmental Management*.

Hendry, K., & Kiel, G. C. (2004). The role of the board in firm strategy: Integrating agency and organisational control perspectives. *Corporate Governance: An International Review, 12*(4), 500-520.

Huang, H., Liu, H., & Yang, B. (2021). Economic policy uncertainty and executive turnover. *China Journal of Accounting Research, 14*(1), 83-100.

Huang, H. J., Habib, A., Sun, S. L., Liu, Y., & Guo, H. (2021). Financial reporting and corporate innovation: a review of the international literature. *Accounting and Finance, 61*(4), 5439-5499.

Jensen, M. C., & Meckling, W. H. (1976). Theory of the firm: Managerial behavior, agency costs and ownership structure. *Journal of financial economics, 3*(4), 305-360.

Jo, H., & Harjoto, M. A. (2011). Corporate governance and firm value: The impact of corporate social responsibility. *Journal of business ethics, 103*, 351-383.

Joecks, J., Pull, K., & Scharfenkamp, K. (2023). Women directors, board attendance, and corporate financial performance. *Corporate Governance: An International Review*.

Jones□Khosla, L., & Gomes, J. (2023). Purpose: From theory to practice. *Global Business and Organizational Excellence*.

Khan, A., Afeef, M., Ilyas, M., & Jan, S. (2023). Does CSR committee drive the association between corporate social responsibility and firm performance? International evidence. *Managerial Finance*.

Khanal, K. (2023). Corporate governance and its impact on financial performance of nepalese commercial banks. *Asian Journal Of Multidimensional Research, 12*(7), 1-13.

Lakonishok, J., Shleifer, A., Vishny, R. W., Hart, O., & Perry, G. L. (1992). The structure and performance of the money management industry. *Brookings Papers on Economic Activity. Microeconomics, 1992*, 339-391.

Liu, Y., Li, J., Liu, G., & Lee, C.-C. (2021). Economic policy uncertainty and firm's cash holding in China: The key role of asset reversibility. *Journal of Asian Economics, 74*, 101318.

Long, S., Zhang, R., & Hao, J. (2022). Asymmetric impact of Sino-US interest rate differentials and economic policy uncertainty ratio on RMB exchange rate. *Journal of International Financial Markets, Institutions and Money, 78*, 101570.

Pamungkas, I. D. (2023). Corporate Governance and Financial Performance on Firm Value: The Case of Indonesia. *WSEAS Transactions on Business and Economics, 20*, 92-103.

Prior, D., Surroca, J., & Tribó, J. A. (2008). Are socially responsible managers really ethical? Exploring the relationship between earnings management and corporate social responsibility. *Corporate Governance: An International Review, 16*(3), 160-177.

Puni, A., & Anlesinya, A. (2020). Corporate governance mechanisms and firm performance in a developing country. *International Journal of Law Management, 62*(2), 147-169.

Qian, L. (2023). Research on the Influence of Economic Policy Uncertainty on Cash Dividend Policy. *Frontiers in Business, Economics and Management, 8*(3), 55-62.

Riyadi, S., Arif, D., Halik, A., & Ariestya. (2023). The effects of board characteristics and firm size on firm value and financial performance. *Accounting, 9*(4), 225-232.

Rusmita, S. A., An-Nafis, M. S. A., Ramadhani, I., & Irfan, M. (2023). The Effect of Good Corporate Governance on Financial Distress in Companies Listed in Sharia Stock Index Indonesia: Machine Learning Approach. In *Advanced Machine Learning Algorithms for Complex Financial Applications* (pp. 220-251): IGI Global.

Saderanta, K. (2022). Kuntouttavan työtoiminnan nuorten asiakkaiden suun terveyden edistäminen: terveysintervention kehittäminen.

Settembre-Blundo, D., González-Sánchez, R., Medina-Salgado, S., & García-Muiña, F. (2021). Flexibility and resilience in corporate decision making: a new sustainability-based risk management system in uncertain times. *Global Journal of Flexible Systems Management, 22*(Suppl 2), 107-132.

Singh, K., & Rastogi, S. (2023). Corporate governance and financial performance: evidence from listed SMEs in India. *Benchmarking: An International Journal, 30*(4), 1400-1423.

Steinfeld, J. M. (2023). Stewardship Theory over Agency Theory. In *Public-Private Stewardship: Achieving Value-for-Money in Public-Private Partnerships* (pp. 123-134): Springer.Szczepankiewicz, E. I., Błażyńska, J., Zaleska, B., Ullah, F., & Loopesko, W. E. (2022). Compliance with Corporate Governance Principles by Energy Companies Compared with All Companies Listed on the Warsaw Stock Exchange. *Energies, 15*(17), 6481.

Tabassum, N., Kaleem, A., & Nazir, M. S. (2013). Impact of real earnings management on subsequent financial performance. *Middle-East Journal of Scientific Research, 17*(4), 551-560.

Van Essen, M., Engelen, P. J., & Carney, M. (2013). Does “Good” corporate governance help in a crisis? The impact of country□and firm□level governance mechanisms in the E uropean financial crisis. *Corporate Governance: An International Review, 21*(3), 201-224.

Wang, M.-J., Su, X.-Q., Wang, H.-D., & Chen, Y.-S. (2017). Directors’ education and corporate liquidity: evidence from boards in Taiwan. *eview of Quantitative Finance and Accounting, 49*, 463-485.

Zhang, L., Chen, W., & Hu, N. (2023). Economic policy uncertainty and stock liquidity: evidence from China. *International Journal of Emerging Markets, 18*(1), 22-44.
