# Supplementary material for: Corporate governance, financial performance, and economic policy uncertainty. Evidence from emerging Asian economies
Source: PLoS One. 2024 Dec 5;19(12):e0312292. doi: 10.1371/journal.pone.0312292 (PMC11620350; doi:10.1371/journal.pone.0312292)
Supplement: Attachment — Submitted filename: Responce to Reviewer.docx [file pone.0312292.s004.docx]

**Reviewer #1:**

I writed my notes on file. Its contributions to policy makers and literature is not clear and weakly. Sample is not enough. Authors comments must be depend on result of analyse. Comments are very generally. Some comments are weak and not depent on literature.

**Response to reviewer 1:** Thank you for the review and suggested me your significant feedback. I corrected all the suggestions recommended through note file in the main manuscript. Please find Yellow Highlighted in manuscript for the track changes for each points. The policy implications make clearer and more innovative. The literature review are make more accurate with recent references insight. All the arguments in the results make effective based on authors own findings conclusion and its justification to previous study literature. The following correction are done according to suggestions.

1. The abbreviation of EPS properly used. Please find page 1 Abstract and line# 13 yellow highlighted.
2. The references missing in introduction cited. (Please find page 2 Line 33 and 41)
3. The Week statement in the Introduction removed (Please find page 2 and yellow highlighted)
4. Results implications are written at page 3-line 86-93 yellow highlighted.
5. In Methodology section, unnecessary information removed. ( Please find page 8)
6. The reason for the selecting non-financial firms are given at page 8-9 yellow highlighted. Moreover, reason of selection of these economies are given.
7. GDP spelling mistakes corrected.
8. The reason for the use of GMM are given at page 12 and line #327-343.
9. The outlier in the Table 2 Descriptive statistics corrected.
10. The AR (1) and AR (2) value in GMM analysis are given at page 18 yellow heighted in the Table.
11. In Conclusion section general comments are removed.
12. The policy implication is clearly mentioned with respect to government, investors, legislators and investors. Please find page 22 and line #521-531 and yellow highlighted.

**Reviewer #2:**

The manuscript entitled "Corporate Governance, Financial Performance and Economic Policy Uncertainty: Evidence From Emerging Asian Economies" investigates an important subject. The following comment could enhance the level of the work:
1. The introduction could be strengthened by providing a more concise and focused explanation of the research gap and objectives.

**Response to point 1:** Research gap and objectives are clearly mentioned in introduction section. The background is also discussed according to the gaps and objectives. Please find page 2-3 from Line number 50-63 for track changes of the Gaps and Line # 64-71 for the Objectives.

2. Consider integrating more recent references to enhance the currency of the literature review. see for example:
Abdullah, H., & Tursoy, T. (2023). The Effect of Corporate Governance on Financial Performance: Evidence From a Shareholder-Oriented System. Interdisciplinary Journal of Management Studies (Formerly known as Iranian Journal of Management Studies), 16(1), 79-95. doi: 10.22059/ijms.2022.321510.674798

**Response to point 2:** The latest references added in the literature with critical review. Please find page 4-7 from Line# 99-214 green highlighted.

3. The theoretical framework is well-developed, drawing upon various theories such as agency theory, stewardship theory, and stakeholder theory. However, it would be beneficial to provide a clearer explanation of how these theories inform the hypotheses.

**Response to point 3:** The theories are discussed in relation to the hypothesis estimated. On the theoretical base we develop the hypothesis. Please find page 4 and line # 103-119 green highlighted for the track changes.

4. Ensure that each hypothesis is clearly stated and aligned with the theoretical underpinnings.

**Response to point 4:** The hypothesis are developed on the ground of the underpinning theories and the empirical literature.

5. Provide more details on the specific variables used to measure corporate governance, financial performance, and economic policy uncertainty. what is DGP? do the authors mean GDP?

**Response to point 5:** More detail on variables are added according to suggestion. Please find page 10 and green highlighted in variable measurement section for the track changes. The “GDP” corrected spelling mistake.

6. Clarify the rationale for selecting the generalized method of moments (GMM) for robustness analysis. How could you calculate Adjusted R square for GMM in table 4? How could the correlation between ROA and ROE be 1? see: Abdullah, H., Tursoy, T. Capital structure and firm performance: evidence of Germany under IFRS adoption. Rev Manag Sci 15, 379–398 (2021). <https://doi.org/10.1007/s11846-019-00344-5>

**Response to point 6:** The rationale of selecting GMM for robustness analysis are given according to suggestions (Please find page 12 and line #327-343 and green highlighted for track changes). Moreover, the table 4 represents the regression Table that includes the Adjusted R square but GMM included the AR(1) and AR(2) values in Table 5. The Outlier of the correlation resolved.

7. Discuss any potential limitations of the methodology, such as data availability or sampling bias.

**Response to point 7:** The limitations make wider related to methodology, such as data availability or sampling. Please find page 22-23 and line #332-345 and green highlighted.

Thank You
