# Supplementary material for: Corporate governance, financial performance, and economic policy uncertainty. Evidence from emerging Asian economies
Source: PLoS One. 2024 Dec 5;19(12):e0312292. doi: 10.1371/journal.pone.0312292 (PMC11620350; doi:10.1371/journal.pone.0312292)
Supplement: Attachment — Submitted filename: Responce to reviewers commenst Vick.docx [file pone.0312292.s005.docx]

**Reviewer #2**

 The authors followed my comments all. So, the manuscript is not publishable from my perspective.

**Response to Reviewer 2:** Thank you for the review. In the last version, I correct all your suggestions.

**Reviewer #3**

#1: My main concern is the justification and supporting resources of choosing the three countries, Pakistan, China and Malaysia as comparison. For example Pakistan chosen for its unique governance, China for transitioning to market-orientation, and Malaysia for its robust regulatory environment. Where is the further proof or justifications for these?

**Response to Reviewer Point 1**:

The proper justification are given in introduction section and also in methodology section according to the suggestion. Please find page 3 and yellow highlighted as “Pakistan's relatively nascent corporate governance framework often struggles with enforcement, which hinder financial performance, particularly during periods of high economic policy uncertainty (Shaikh, Khoso, & Jummani, 2024). Similarly, China with more centralized governance structures and state-owned enterprises, demonstrates a different dynamic where government policies and interventions mitigate some uncertainty but may also stifle market-driven growth (Cao, Prior, Gu, & Giurco, 2023). In contrast, Malaysia exhibits a more developed corporate governance system that fosters better financial performance in the face of uncertainty, with robust regulatory frameworks allowing for greater market resilience (Y. Wu & Tham, 2023). The influence of economic policy uncertainty in each country is shaped by the strength of institutions and governance practices, impacting corporate outcomes differently. Therefore, this study extracts a gap in literature to investigate the corporate governance along with the economic policy uncertainty effect the financial performance in different culture that provide strong evidence of both strong and week corporate governance structure. Similarly, this study is also important for improving the corporate sector in the emerging economy where corporate governance mechanism is week.”

#2: Accordingly, there is no discussion about the comparison between the three countries, which is not aligned to the claimed contribution.

**Response to Reviewer Point 2:** The discussion on comparison are written according to suggestions with references, Please find page 3 yellow highlighted and page 8-9 yellow highlighted for track changes.

#3: Sentences in page 3 and page 22 are repeated verbatim, regarding the contribution of the study.

**Response to Reviewer Point 3:** The repeating words deleted.

#4: Referring to these sentences, the second contribution is missing. From first then after that was the third.

**Response to Reviewer Point 4:** In contribution paragraph, the refered objective corrected. Please find page 3 yellow highlighted for track changes.
#5: What is CSR Suitability Committee? Are you trying to actually state 'Sustainability' instead of 'suitability'?

**Response to Reviewer Point 5:** It is CSR committee existence that corrected according to suggestions in the manuscript.
#6: Does the item CSR committee, covers Sustainability Committees as well? What if the committee is combined with another committee and not stand alone? Is that also included?

**Response to Reviewer Point 6:** It is CSR committee existence that corrected according to suggestions in the manuscript.
#7: The abbreviations used for the items in your results tables for example ACI, AGE, BS, etc were not spelled out anywhere in the text. Some were, but some not. The reader may make a guess based on the conceptual framework but that is not appropriate and may be inaccurate and subjective.

**Response to Reviewer Point 7:** Abbreviation make corrected according to suggestions. Please find page 11 yellow highlighted and also under the table of descriptive statistics for first uses.
#8: Assuming that my concern in #1 and #2 is addressed, and that it turns out that the choice of the 3 countries are reasonable, justified and has significant impact, I have no further comments on the method and data analysis used.

**Response to Reviewer Point 8:** The selections of three countries are given in Introduction and also in methodology.
